# H/KDEL receptors mediate host cell intoxication by a viral A/B toxin in yeast

**DOI:** 10.1038/srep31105

**Published:** 2016-08-05

**Authors:** Björn Becker, Andrea Blum, Esther Gießelmann, Julia Dausend, Domenik Rammo, Nina C. Müller, Emilia Tschacksch, Miriam Steimer, Jenny Spindler, Ute Becherer, Jens Rettig, Frank Breinig, Manfred J. Schmitt

**Affiliations:** 1 Molecular and Cell Biology, Department of Biosciences (FR 8.3) and Center of Human and Molecular Biology (ZHMB), Saarland University, D-66123 Saarbrücken, Germany; 2 Institute of Physiology, Saarland University, D-66421 Homburg, Germany

## Abstract

A/B toxins such as cholera toxin, *Pseudomonas* exotoxin and killer toxin K28 contain a KDEL-like amino acid motif at one of their subunits which ensures retrograde toxin transport through the secretory pathway of a target cell. As key step in host cell invasion, each toxin binds to distinct plasma membrane receptors that are utilized for cell entry. Despite intensive efforts, some of these receptors are still unknown. Here we identify the yeast H/KDEL receptor Erd2p as membrane receptor of K28, a viral A/B toxin carrying an HDEL motif at its cell binding β-subunit. While initial toxin binding to the yeast cell wall is unaffected in cells lacking Erd2p, binding to spheroplasts and *in vivo* toxicity strongly depend on the presence of Erd2p. Consistently, Erd2p is not restricted to membranes of the early secretory pathway but extends to the plasma membrane where it binds and internalizes HDEL-cargo such as K28 toxin, GFP^HDEL^ and Kar2p. Since human KDEL receptors are fully functional in yeast and restore toxin sensitivity in the absence of endogenous Erd2p, toxin uptake by H/KDEL receptors at the cell surface might likewise contribute to the intoxication efficiency of A/B toxins carrying a KDEL-motif at their cytotoxic A-subunit(s).

Yeast killer toxin K28 is an α/β heterodimeric protein toxin that is naturally secreted by virus-infected killer strains of the yeast *Saccharomyces cerevisiae*. During *in vivo* intoxication, K28 enters sensitive cells in a two-step receptor-mediated process in which the toxin crosses two major barriers, the yeast cell wall and the cytoplasmic membrane, followed by retrograde transport through the secretory pathway guided by a C-terminal HDEL motif and putative ER targeting signal at the toxin’s cell binding B/β-subunit. After ER exit and entrance into the cytosol the toxin dissociates into its subunit components and kills through its α-subunit by blocking nuclear DNA synthesis and arresting cells at the G1/S boundary of the cell cycle (Fig. 1)^1^,^2^,^3^,^4^,^5^. The initial step in this receptor-mediated process of host cell invasion and killing involves toxin binding to cell wall mannoproteins that are utilized as primary K28 receptors. Mutations in chromosomal genes (e.g. *mnn2*, *mnn5*) affecting α-1,3-mannotriose side-chain structure in cell wall mannoproteins cause K28 toxin resistance in whole cells, whilst spheroplasts retain normal toxin sensitivity^6^,^7^. Since toxin resistance in yeast cell wall mutants is saturable and leaky at higher toxin concentration, alterations in mannoprotein side-chain structure do not prevent residual cell wall binding affinity of the toxin^8^. Rather, toxin binding to the outer yeast cell surface is presumed to ensure toxin access to a secondary receptor at the level of the plasma membrane responsible for subsequent endocytotic internalization of the toxin^2^. Although this K28 membrane receptor has not yet been identified, the following findings reasoned us to speculate that it might be the cellular H/KDEL receptor Erd2p: (i) Whole cells and spheroplasts of a yeast ∆*erd2* knock-out mutant lacking Erd2p are toxin resistant and impaired in toxin internalization; (ii) mutant K28 toxin lacking its β-C-terminal HDEL motif is non-toxic and incapable to enter cells^2^,^9^. While the HDEL motif and putative ER targeting signal of K28 is part of the toxin’s cell binding β-subunit involved in retrograde toxin trafficking to the ER, KDEL-like motifs in A/B toxins such as cholera toxin, *Pseudomonas* exotoxin A and the heat-labile toxins (HLT) of *E*. *coli* are present at the cytotoxic A/α-subunit(s)^10^,^11^ (Fig. 1); so far, however, these motifs have not been associated with a function in toxin cell entry. Based on the striking and frequent occurrence of KDEL-like motifs in microbial A/B toxins and the pronounced importance of such a motif for K28 *in vivo* toxicity, we focused our attention on the yeast HDEL receptor Erd2p as potential plasma membrane receptor of K28.

## Results

### Erd2p mediates toxin binding and uptake in yeast spheroplasts

The pivotal role of the yeast H/KDEL receptor Erd2p in host cell intoxication is illustrated by the conference of complete K28 resistance of a ∆*erd2* mutant lacking Erd2p (Fig. 2A). While this phenomenon was originally attributed to its function as retrieval receptor during retrograde toxin transport to the ER^2^, we now identify a strict correlation between *ERD2* copy number, toxin binding to yeast spheroplasts and overall host cell sensitivity, portraying the central role of Erd2p in K28 toxicity. While toxin binding to whole cells is not negatively affected in an ∆*erd2* mutant^12^ (data not shown), toxin binding to spheroplasts from cells lacking Erd2p (∆*erd2*) is severely impaired and became detectable only at higher cell concentrations (Fig. 2B), most likely reflecting K28 binding to remnants of interconnected cell wall β-1,3/1,6-glucans, mannoproteins and chitin that are not entirely removed during enzymatic spheroplast formation as previously reported^13^,^14^ and confirmed here by calcofluor white staining of remaining cell wall chitin in yeast spheroplasts generated by zymolyase treatment (Supplementary Figure S1). However, besides K28 binding to cell wall remnants in yeast spheroplasts, low affinity toxin binding to some other cell surface component, independent of Erd2p, cannot be excluded. Interestingly, toxin binding deficiency of ∆*erd2* spheroplasts could be gradually restored by a stepwise increase in Erd2p expression, finally resulting in a hypersensitive phenotype after multi-copy expression (Fig. 2A,B). Consistent with the observed decrease in toxin binding to ∆*erd2* spheroplasts, also toxin internalization was strongly reduced in the absence of Erd2p (Fig. 2C), indicating that H/KDEL receptors are critically involved in the endocytotic uptake of K28 from the cell surface. Notably, the minor amount of internalized toxin detectable in *∆erd2* cells is not sufficient to confer *in vivo* toxicity (Fig. 2A) and, therefore, likely caused by receptor-independent endocytosis events which target the toxin to vacuolar/lysosomal degradation; a phenomenon that is also assumed to occur during A/B toxin invasion of mammalian cells^15^,^16^.

The importance of Erd2p in toxin cell binding and internalization from the plasma membrane in conjunction with the central role of the toxin’s HDEL motif for *in vivo* toxicity strongly point towards a function of Erd2p at the cell surface. In such a case, exogenously applied proteins carrying a HDEL motif should be bound and internalized by H/KDEL receptors at the plasma membrane. We therefore analyzed *in vivo* uptake of GFP in the presence or absence of a C-terminal HDEL sequence after cell fractionation of wild-type spheroplasts. As illustrated in Fig. 2D, GFP^HDEL^ was efficiently internalized and detectable in the endosomal as well as in the cytosolic fraction, whilst unmodified GFP was incapable to enter cells and entirely localized in the P13 fraction, resembling a mixture of plasma membrane, Golgi and ER membranes, as well as cell wall remnants that are not completely removed during enzymatic spheroplast formation. Thus, attachment of an HDEL sequence to GFP is necessary and sufficient to ensure its endocytotic uptake and strongly implies that Erd2p is responsible for HDEL-cargo internalization from the cell surface.

We next asked if purified and extracellularly applied Kar2p (BiP) as natural Erd2p ligand and essential ER chaperone can be taken up from the cell surface and, thereby, restore cell growth of a yeast *kar2*^ts^ mutant at the restrictive temperature (Fig. 3A,B). In the corresponding experiments, cell growth of a yeast *kar2*^ts^ mutant was assessed by measuring dissolved oxygen consumption in the culture medium with an oxygen-sensitive fluorescence sensor. In contrast to *kar2*^ts^ cells which did not grow at the restrictive temperature in the presence of a negative control protein, cell cultivation in the presence of increasing amounts of Kar2p resulted in a dose-dependent rescue of the growth defect of the *kar2*^ts^ mutant (Fig. 3C). Under identical experimental conditions, extracellular addition of Kar2p to spheroplasts of HDEL receptor defective ∆*erd2* cells did not improve cell growth in general (Supplementary Figure S2), indicating that Kar2p addition is unlikely to rescue *kar2*^ts^ mutant cells through extracellular functions but rather acts through Erd2p-mediated HDEL-cargo internalization from the cell surface. To proof this hypothesis and demonstrate Kar2p internalization biochemically, spheroplasts from wild-type cells and from the ∆*erd2* knock-out mutant were treated with equal amounts of purified Kar2p for 2 h at 30 °C, repeatedly washed to remove remaining HDEL-cargo from the cell surface, and subsequently lysed and subjected to SDS-PAGE and immunoblot. As illustrated in Fig. 3D, internalized Kar2p was clearly detectable in the lysate from wild-type spheroplasts while no such signal was seen in ∆*erd2* cells lacking Erd2p, confirming that the yeast HDEL receptor is indeed capable to bind and internalize HDEL-cargo from the plasma membrane.

### H/KDEL receptor colocalization at the plasma membrane

Intracellular localization of a fluorescent and *in vivo* functional receptor variant (Erd2p-GFP), either expressed from its natural promoter and chromosomal *ERD2* locus or placed under transcriptional control of *GAL1* on a multi-copy plasmid, confirmed the documented colocalization of H/KDEL receptors with the Golgi marker Anp1p^17^. In both cases, however, fluorescent patches of Erd2p-GFP were also present at the cell periphery and partially colocalized with markers of the plasma membrane (Can1p) and eisosomes (Sur7p, Pil1p) (Fig. 4A,B). The presence of Erd2p at the cell surface in conjunction with eisosomal proteins indicates that Erd2p can be internalized by endocytosis, a prerequisite for the delivery of HDEL-cargo to the ER and supported by the observed increase in plasma membrane localization of Erd2p in endocytosis defective cells of a yeast Δ*end3* mutant (Fig. 4A). To address this aspect in more detail, we tracked the endocytotic uptake of FM4-64 and demonstrated colocalization of single Erd2p-GFP foci at the cell periphery and in endosomal vesicles derived from the plasma membrane (Fig. 4C). Mobility of Erd2p-GFP vesicles was further assessed by TIRF microscopy and caging diameter (CD) analysis in K28-resistant *end3* cells which are defective in endocytosis and hereby blocked in toxin uptake^2^,^18^,^19^. As expected, Erd2p-GFP vesicles showed a significant decrease in mobility in the *end3* background compared to wild-type as indicated by an increase in the number of small CD corresponding to complete immobility (Fig. 4D). A strong decrease in CD values at 50% was also observed in *end3* cells, consistent with the illustrated immobilization of Erd2p-GFP in this background. Since the only known function of End3p is its participation in endocytosis^20^, these results likewise strongly support that Erd2p is internalized from the plasma membrane.

To biochemically address plasma membrane localization of Erd2p, cell surface biotinylation was performed on wild-type yeast expressing a functional, C-terminal 3xFlag-tagged receptor variant (Erd2p-3xFlag) from its endogenous promoter and natural chromosomal locus, followed by an avidin pull-down to detect Erd2p levels at the cell surface. Although biotinylation of plasma membrane proteins in yeast is widely assumed to be hampered by the cell wall barrier and has so far been restricted to cell wall proteins^21^,^22^, we succeeded to detect Erd2p in the biotinylated cell surface fraction at a level corresponding to about 8.8 ± SD = 0.7% (n = 3) of its total cellular amount (Fig. 5A), nicely matching the amount of plasma membrane localized KDEL receptors (Erd2.1) in mammalian cells^20^.

### Erd2p can function as transport vehicle to deliver RAS to the plasma membrane

To further support the observed cell surface localization of the yeast HDEL receptor, a physiological read-out originally developed^23^ to identify protein interactions at the plasma membrane was adapted to analyze if Erd2p is capable to function as intracellular transport vehicle for the delivery of RAS to the plasma membrane (Fig. 5B). In this assay, RAS-mediated cAMP signaling can only be restored and enable growth of a yeast *cdc25-2*^ts^ mutant when Erd2p delivers a constitutively active variant of mammalian RAS (mRAS) lacking its natural farnesyl membrane anchor to the plasma membrane (Fig. 5C)^24^,^25^. Based on the *in vivo* topology of H/KDEL receptors in yeast, plant and mammalian cells^26^,^27^,^28^, a fusion of mRAS to the C-terminus of Erd2p should expose mRAS to the cytosolic face of the plasma membrane (Fig. 5C). To exclude false positive activation of adenylate cyclase (Cyr1p) in close proximity of the plasma membrane, an additional control was included by fusing mRAS to the cytosolic C-terminus of Ice2p, an integral membrane protein that exclusively localizes to the cortical and perinuclear ER^27^. As illustrated in Fig. 5D, only expression of Erd2p-mRAS restored cell growth of the *cdc25-2*^ts^ mutant while neither Ice2p-mRAS nor non-fused mRAS had any effect. Potential Cyr1p activation from the Golgi was excluded by using the Golgi membrane marker Emp47p^29^,^30^ as additional control which, after fusion with its cytosolic C-terminus to mRAS, was incapable to restore cell growth of the *cdc25*^ts^ mutant (data not shown).

Taken together, these data highlight the importance of Erd2p in the internalization of K28 toxin and HDEL-bearing cargo from the cell surface and, thereby, identify a novel function of H/KDEL receptors at the plasma membrane. Interestingly, all three mammalian KDEL receptors (Erd2.1–Erd2.3) were capable to complement the growth defect of a yeast ∆*erd2* knock-out and restored K28 toxin sensitivity in the absence of endogenous Erd2p to levels comparable to those after complementation by the yeast receptor Erd2p (Fig. 5E,F). As mammalian KDEL receptors were likewise most recently shown to localize at the cell surface^31^,^32^, one might speculate that H/KDEL receptors at the plasma membrane contribute to the overall intoxication efficiency even in A/B toxins which carry a KDEL-like motif at their non-cell binding A-subunit(s).

## Discussion

Until recently, the main function of cellular H/KDEL receptors was primarily seen in the retrieval and retrograde transport of soluble ER residents from the Golgi back to the ER^33^. With respect to the intracellular localization of mammalian KDEL receptors, Erd2.1 was originally reported to reside in Golgi and Golgi/ER intermediate compartments^34^, while ligand-dependent ER redistribution was demonstrated for Erd2.2^33^. Besides the well documented function of H/KDEL receptors in Golgi/ER protein retrieval, more recent studies indicate additional functions in cellular signalling and development, in T-cell homeostasis as well as in controlling viral infections^35^,^36^. In the present study we extend these functions by identifying an essential role of H/KDEL receptors at the yeast cell surface in cargo binding and internalization during intoxication by a killer toxin (K28) carrying a potential ER-targeting signal (HDEL) at its cell binding B/β-subunit.

During host cell invasion by K28, receptor endocytosis represents the initial critical step as it ensures that the toxin can pass the plasma membrane by a series of events including clathrin and AP2 mediated endocytosis followed by retrograde toxin transport to the ER^2^,^12^. We now detail these findings by showing that a minor but significant fraction of the yeast H/KDEL receptor is also present at the plasma membrane where it mediates binding and endocytotic internalization of HDEL-cargo. While receptor-deficient Δ*erd2* cells are unaffected in toxin binding to the yeast cell wall^12^, we here demonstrate that toxin binding to spheroplasts is severely impaired but can be fully restored by a stepwise increase in Erd2p copy number, resulting in a significant increase in toxin spheroplast binding and a corresponding increase in K28 sensitivity. Interestingly, the optimal pH for K28 *in vivo* killing is in the range of pH 4.7 to 5.8 which perfectly matches the mildly acidic pH of the Golgi at which H/KDEL receptors have been demonstrated to show maximal binding of H/KDEL-ligands while cargo release occurs at the neutral pH of the ER lumen^37^,^38^. Consistent with the proposed function of Erd2p in cargo uptake from the cell surface, a C-terminal HDEL extension of GFP is needed and sufficient for cellular uptake, and exogenously applied HDEL-cargo such as Kar2p (BiP) can be internalized *in vivo* and is targeted to the ER where it restores cell growth of a *kar2*^ts^ mutant in the absence of endogenous Kar2p. Thus, recognition and binding of H/KDEL-ligands to cargo receptors at the plasma membrane, shown here for Kar2p, GFP^HDEL^ and K28 toxin, is sufficient to trigger endocytotic uptake. Additional TIRF microscopy of cells expressing an *in vivo* functional green fluorescent receptor variant confirmed that Erd2p-GFP signals appear in punctuated clusters at the plasma membrane that can be tracked and allocated to endocytic vesicles, strongly supporting that Erd2p is present in endosomes derived from the plasma membrane. In fact, the observed uptake of HDEL-cargo from the cell surface and enhanced presence of Erd2p at the cell surface in endocytosis defective Δ*end3* mutant cells suggests a physiological function of plasma membrane localized H/KDEL receptors in ensuring internalization of cargo that has failed ER retention. Due to the limited capacity of the cellular ER retention system, receptor-mediated uptake of leaky chaperones and KDEL-bearing cargo can be assumed to be an intrinsic mechanism of protein retrieval at the level of the plasma membrane. Such mechanism could ensure cell viability under normal and under stress conditions and would be consistent with recent reports on KDEL receptor localization and cargo clustering at the mammalian cell surface^32^. With respect to the plant A/B toxin ricin it is interesting to note that its cytotoxic A chain (RTA) extended by a C-terminal HDEL or KDEL motif becomes toxic for mammalian cells even in the absence of its natural cell binding B-subunit^31^, suggesting that KDEL receptors at the mammalian cell surface might also contribute to the overall intoxication efficiency of A/B toxins whose KDEL-like motif is present at the cytotoxic A-subunit(s).

**Figure 1:**
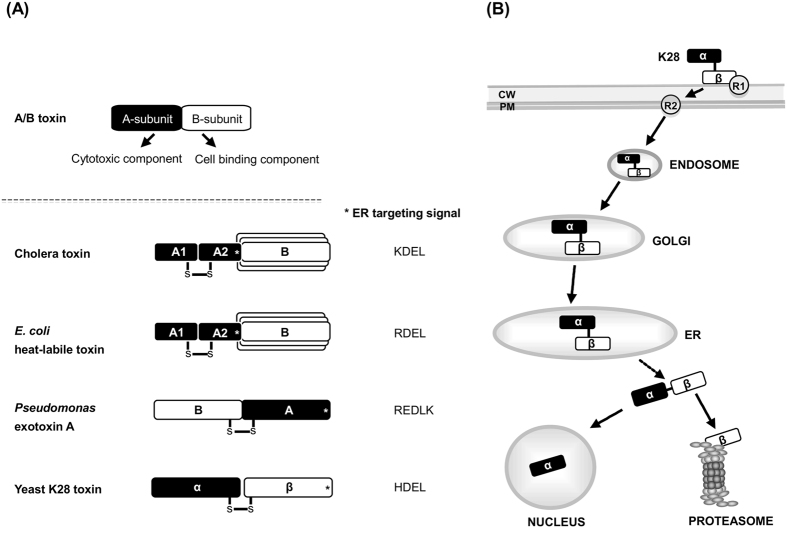
(**A**) Schematic outline of the general structure of microbial and viral A/B toxins carrying a C-terminal KDEL-like motif and potential ER targeting signal. (**B**) Host cell intoxication of yeast killer toxin K28 via receptor-mediated endocytosis, retrograde trafficking through the secretory pathway, and final killing in the nucleus (R1, cell wall receptor utilized by K28; R2, plasma membrane receptor for K28 uptake); adapted and extended from refs 15 and 5.

**Figure 2:**
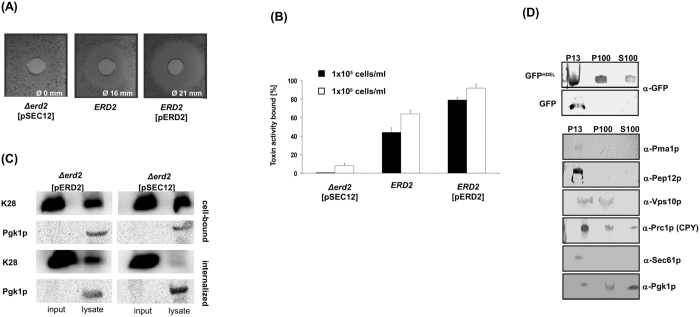
Erd2p-mediated toxin binding and cargo uptake in yeast spheroplasts. (**A**) K28 phenotype of cells lacking Erd2p (∆*erd2* [pSEC12]) or expressing Erd2p in single copy (*ERD2*) or multi copy (*ERD2* [pERD2]). (**B**) Toxin binding to spheroplasts in dependence of cell concentration and Erd2p copy number. Each experiment was performed in triplicate (n = 3) on spheroplasts treated with K28 toxin (1 μg/ml), shown is the mean average ± SD. (**C**) Immunoblot of the amount of cell-bound and internalized K28 toxin in lysates of Δ*erd2* cells expressing wild-type Erd2p (pERD2) or Sec12p as negative control (pSEC12) after treatment with K28 toxin (3 μg/ml). Relative amount of internalized toxin was determined after proteinase K treatment and removal of cell bound toxin; phosphoglycerate kinase (Pgk1p) served as cytosolic marker. (**D**) Cell fractionation of wild-type spheroplasts treated with purified GFP or GFP^HDEL^ (2 μg/ml each) and probed with the indicated antibodies (P13 = 13,000 g pellet; P100 = 100,000 g pellet; S100 = 100,000 g supernatant). Cropped blots shown in (**C**,**D**) originate from the same gels and were thus run under the same experimental conditions.

**Figure 3:**
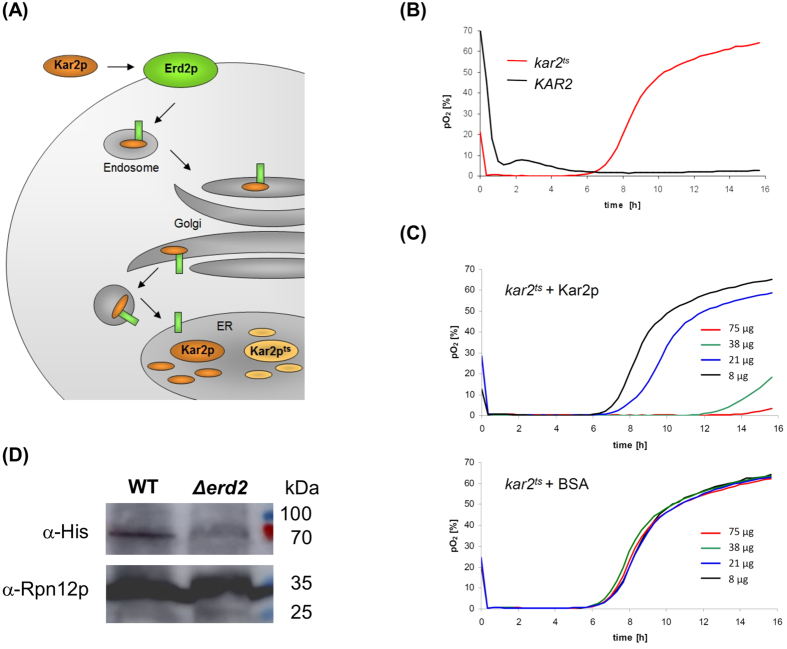
(**A**) Experimental setup to analyze Erd2p-mediated uptake and retrograde transport of the HDEL-cargo Kar2p into the ER of a yeast *kar2*^ts^ mutant. (**B**) Dissolved oxygen content in the culture supernatant of spheroplasts from *KAR2* wild-type and *kar2*^*ts*^ cells at 37 °C. (**C**) Dissolved oxygen content in the culture supernatant of spheroplasts from *kar2*^*ts*^ cells (37 °C) in the presence of increasing amounts of Kar2p or bovine serum albumin (BSA, negative control). Each experiment in (**B**,**C**) was performed in triplicate (n = 3); shown is the mean average. (**D**) Immunoblot of internalized Kar2p (containing an N-terminal (His)_6_ tag and detected with anti-His) in cell lysates of spheroplasts from wild-type strain SEY6210 and HDEL receptor defective Δ*erd2* cells after treatment with recombinant Kar2p (9 μg/ml) for 2 h at 30 °C. Rpn12p, a regulatory subunit of the 26S proteasomal lid, served as loading control. Cropped blots shown in (**D**) originate from the same gel/blot after stripping and antibody reprobing.

**Figure 4:**
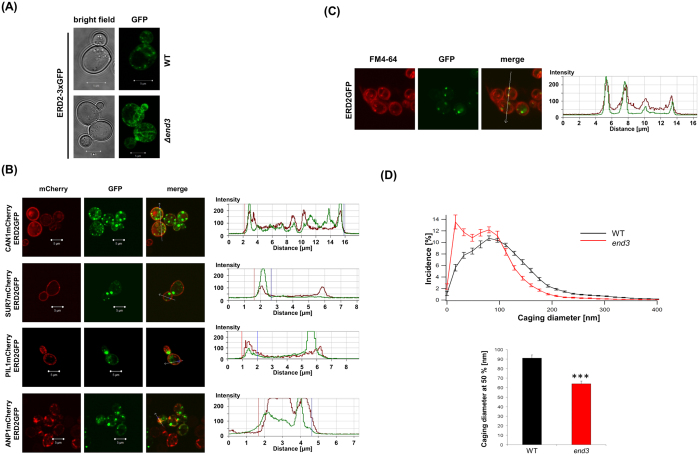
(**A**) Intracellular localization of Erd2p-3xGFP expressed from the chromosomal *ERD2* locus and endogenous promoter in wild-type (WT) and in Δ*end3* mutant cells defective in endocytosis. (**B**) Wild-type cells coexpressing Erd2p-GFP and red fluorescent (mCherry) marker proteins of the plasma membrane (Can1p), eisosomes (Sur7p, Pil1p) and Golgi (Anp1p). (**C**) Colocalization of Erd2p-GFP with FM4-64. (**D**) Mobility of Erd2p-GFP vesicles in *end3* and wild-type cells (WT) determined by TIRF microscopy and displayed as normalized CD distribution histogram (N = 15, n = 180 cells). CD histograms were integrated and CD values reached by 50% of all CDs were calculated (***p < 0.001). Mean average and standard deviation is displayed.

**Figure 5:**
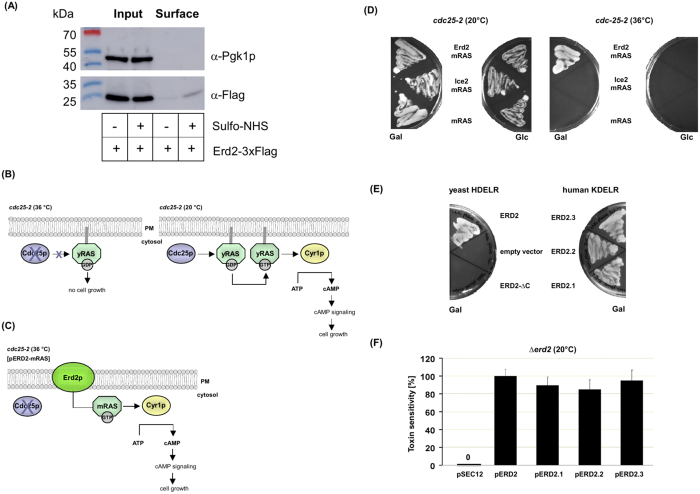
(**A**) Cell surface biotinylation of yeast Erd2p. Wild-type cells expressing Erd2p-3xFlag from its endogenous promoter and chromosomal locus were biotinylated by treatment with (+) or without (−) Sulfo-NHS-SS-Biotin and purified with avidin beads. Whole cell lysates (input) served as control to determine the total amount of Erd2p (detected with anti-Flag), while phosphoglycerate kinase (Pgk1p) served as cytosolic marker protein to determine cellular integrity of the samples. Membrane fraction (surface) represents the pool of Erd2p at the cell surface (note that the faint signal in the surface fraction without Sulfo-NHS treatment results from unspecific binding of Erd2-3xFlag to the avidin-coupled agarose beads). (**B**,**C**) Schematic outline of the RAS recruitment system. Expression of a constitutively active cytosolic variant of mammalian RAS (mRAS) lacking a farnesyl membrane anchor activates adenylate cyclase (Cyr1p) and restores cell growth of a *cdc25-2*^ts^ mutant only when fused to a membrane protein vehicle (Erd2p) that ensures its delivery to the plasma membrane. (**D**) Growth phenotype of a yeast *cdc25-2*^ts^ mutant expressing cytosolic mRAS or mRAS fusions to the cytosolic C-termini of Ice2p (Ice2p-mRAS) or Erd2p (Erd2p-mRAS). (**E**) Human KDEL receptors complement Erd2p function in yeast. Growth of Δ*erd2* cells expressing either of the three human KDEL receptors (ERD2.1, ERD2.2, ERD2.3), a full-length (ERD2) or a C-terminal truncated and, thus, non-functional yeast H/KDEL receptor (ERD2-∆C), or empty vector control cells monitored under inducing conditions after 5 d at 30 °C. (**F**) Human KDEL receptors restore K28 toxin sensitivity in a yeast Δ*erd2* null mutant. Sensitivity of Δ*erd2* cells expressing yeast Erd2p was set 100%. Mean average (n = 10) and standard deviation is displayed. For p-value calculation, cells expressing Erd2p were compared to cells expressing the indicated mammalian KDEL receptor.

In sum our data highlight the importance of H/KDEL receptors in cargo binding and internalization and, thus, identify a novel function of Erd2p at the yeast cell surface. Based on the data presented here, the general model of K28 internalization (see Fig. 1) can be refined by an initial interaction with host cell H/KDEL receptors at the plasma membrane level. The presence of Erd2p at the cell surface might also explain - albeit not yet mechanistically - why H/KDEL-bearing A/B toxins, once internalized, are subsequently sorted to the protecting environment of the secretory pathway rather than being recognized as substrate for lysosomal targeting and degradation. In addition to mediating A/B toxin uptake and ensuring recovery of inadvertently secreted KDEL-cargo from the cell surface, KDEL receptors might also participate in signalling from the cell surface, similar to their recently described role in signalling within the Golgi complex^39^,^40^. In future experiments we will address this aspect to gain mechanistic insight into the full range of H/KDEL receptor functions at the eukaryotic cell surface.

## Materials and Methods

### Cultivation and staining of yeast cells

*S*. *cerevisiae* strains used in this study and listed in Supplementary Table S1 were routinely grown at 30 °C in standard YPD complex, synthetic complete (SC) or d/o media containing 2% glucose or 3% galactose. Temperature sensitive *cdc25-2*^ts^ mutants were grown at the permissive temperature (20 °C) and shifted to the restrictive temperature (36 °C) when assayed for cell growth complementation^23^. Yeast transformation and generation of spheroplasts was performed as previously described^7^. Remnant cell wall components in yeast spheroplasts generated by zymolyase treatment were detected by calcofluor white (CFW) staining. In brief, whole yeast cells or spheroplasts were incubated in a ready to use CFW staining solution (Fluka, 1/10 dilution in 1 M sorbitol-stabilized incubation buffer [pH 4.7]) for 1 min at room temperature and immediately analyzed under a fluorescence microscope (Keyence BioZero-8000K) using CFW standard settings.

### Vector construction for KDELR expression

Wild-type Erd2p C-terminally extended by yeast enhanced yGFP was constructed by SOE-PCR^41^. All other constructs were amplified by conventional PCR with primers as listed in Supplementary Table S2. For the construction of EMP47mRAS, a synthetic DNA sequence of *EMP47* (GeneArts, ThermoScientific, Appendix S1) was integrated via *Xho*I/*Spe*I into pRS316mRAS. Yeast expression constructs resemble single-copy centromeric vectors based on pRS316 or pRS315 which were modified by integration of a *Not*I/*Sac*I fragment including a *GAL1* promoter and a *CYC1* terminator^42^. Constructs of ERD2, ERD2GFP, EMP47mRAS and ICE2mRAS were expressed from pRS316, mCherry fusions were cloned into pRS315. cDNAs of human KDEL receptors were subcloned via *EcoR*I/*Sal*I into pRS316 and integrated via *Apa*I/*Sac*I into pRS315. Primers used for PCR amplification are listed in Supplementary Table S2.

### Chromosomal yGFP-tagging of Erd2p via homologous recombination

Triple yGFP-tagging of Erd2p was performed to enhance its detection in live cell imaging experiments. For chromosomal tagging of Erd2p with three C-terminal copies of yGFP, a synthetic DNA sequence (ThermoScientific, GeneArts) containing 200 bp from the 3′-end of *ERD2* (without a stop codon) followed by three copies of yGFP, a stop codon, a transcriptional *ADH1* terminator, a *URA3* selection marker flanked by promoter and terminator sequences and additional 200 bp from the 3′-UTR of *ERD2* was designed as illustrated in Supplementary Figure S3. The DNA construct was linearized with *Eco*RI, isolated and purified from an agarose gel and subsequently used to transform *S*. *cerevisiae* BY4742. Successful homologous recombination was monitored by selecting yeast transformants on ura d/o agar, and positive clones were verified by Western analysis (data not shown) and confocal LS microscopy.

### KDELR complementation and toxin sensitivity/binding analysis

Although a chromosomal deletion of *ERD2* is lethal in yeast, Δ*erd2* knock-out cells can be kept alive by simultaneously co-expressing extra copies of *SEC12* from an episomal 2 μ plasmid encoding the GDP/GTP exchange factor Sec12p required for vesicle budding from the ER^43^. After transformation of Δ*erd2* cells with vectors expressing mammalian (Erd2.1–Erd2.3) or yeast (Erd2p) H/KDEL receptors either from a centromeric or a multi-copy plasmid under transcriptional control of *GAL1*, the 2 μ *SEC12* expression plasmid was eliminated by 5′-FOA selection (leu d/o gal with 0.1% FOA). Cells were grown for 5 d at 30 °C and the ability of each tested H/KDEL receptor to complement Erd2p function was confirmed by cell growth and regain of K28 toxin sensitivity. In brief, K28 sensitivity of strains expressing yeast or mammalian H/KDEL receptors was determined in an agar diffusion assay on methylene blue agar (MBA; pH 4.7) by using an overlay of 10^6^ cells per plate of the corresponding yeast strain^9^. A cell-free concentrated culture supernatant of a K28 killer strain was used as toxin source. In brief, 100 μl of the K28 toxin concentrate (3 μg/ml) were pipetted into 10 mm wells cut into the agar and plates were incubated for 7 d at 20 °C. In each case, toxin sensitivity is expressed by the diameter of the resulting zone of growth inhibition around the well. For toxin binding analysis, yeast spheroplasts (1 × 10^5^ to 1 × 10^6 ^cells/ml) from cultures grown to late exponential phase and resuspended in McIlvaine buffer pH 4.7 containing 0.8 M sorbitol were incubated in the presence of K28 toxin (1 μg/ml) for 60 min at 4 °C. After low-speed centrifugation (300 g), residual toxin activity in the cell free supernatant was determined on MBA plates against the sensitive strain 192.2d. A killing zone diameter of 13 mm corresponds to 1,000 U/ml or 0.1 μg purified K28 toxin^7^.

### Cell surface biotinylation

Yeast cells expressing Erd2p-3xFlag from its endogenous promoter and natural chromosomal locus were grown to exponential phase (OD_600_ = 2), harvested and subsequently used for cell surface biotinylation with a commercial biotinylation kit (Pierce) by using a slightly modified protocol of the manufacturer. In brief, yeast cells were washed three times with cold PBS (pH 7.2) and labelled for 90 min with Sulfo-NHS-SS-Biotin (1 mg/ml in PBS) at 4 °C. The biotinylation reaction was quenched two times for 15 min at 4 °C and the cells were washed three times with cold TBS buffer. Cells were resuspended in 400 μl lysis buffer containing protease inhibitor (Roche) and lysed with glass beads followed by an incubation on ice for 30 min. After centrifugation for 10 min at 15.000 rpm, an aliquot (50 μl) of the cell lysate was removed as input control and the remaining cell lysate (350 μl) was used for pull-down with avidin agarose beads (Pierce). Pull-downs were performed over night at 4 °C with end-over-end rotation. Samples were washed four times with 1 ml of protease inhibitor containing wash buffer, two times with 1 ml SWS buffer (0.1% Triton X-100 in PBS [pH 7.4], 350 mM NaCl and 5 mM EDTA) and two times with 1 ml wash buffer. Washed avidin beads were transferred to a fresh column, washed again with 1 ml wash buffer and eluted for 1 h at room temperature in 350 μl 3 × SDS buffer containing 50 mM DTT and 5% 2-mercaptoethanol. Aliquots of the input and membrane fraction (20 μl each) were used for SDS-PAGE and Western analysis. By using antibodies against phosphoglycerate kinase (Pgk1p), cellular integrity was checked during the labelling step. Anti-Flag antibodies served as positive control to confirm success of Sulfo-NHS-SS-Biotin labelling of Erd2p.

### RAS recruitment system (RRS)

In yeast, cAMP-dependent cell growth requires an interaction of GTP-charged RAS with adenylate cyclase (Cyr1p) at the plasma membrane^24^. The RRS uses a yeast *cdc25-2*^ts^ mutant in which the guanyl nucleotide exchange factor Cdc25p is inactive at 36 °C, rendering endogenous RAS incapable of activating cell growth via cAMP signaling^25^. Wild-type Erd2p was C-terminally fused to a constitutively active variant of mammalian RAS (mRAS) lacking is natural farnesyl plasma membrane anchor. Detection of cell growth after expressing each mRAS reporter was performed as described below. In brief, *cdc25-2*^ts^ cells transformed with the pRS316-ERD2mRAS were grown under inducing conditions at 20 °C, plated onto galactose ura d/o agar and incubated at the restrictive temperature (36 °C) for 5 d. Cells carrying pADNS-JZ-Ras, pRS316-ICE2mRAS or pRS316-EMP47mRAS served as negative control^23^.

### KDEL-cargo uptake and cell fractionation

GFP and GFP^HDEL^ were cloned into pET24-d^(+)^ as *Nde*I/*Eco*RI fragment and expressed in *E*. *coli* after induction with 1 mM IPTG for 3.5 h at 37 °C. Protein solutions in PBS were obtained by sonification. Recombinant GFP or GFP^HDEL^ (2 μg/ml each) were added to 1–5 × 10^7^ yeast cell spheroplasts resuspended in 20 ml incubation buffer (10 mM Tris/HCl pH 4.7, 0.8 M sorbitol, 10 mM CaCl_2_, 10 mM glucose). After 1 h at 25 °C and 100 rpm, cells were harvested, washed and subjected to mechanical disruption and cell fractionation as previously described^44^. Briefly, cells were resuspended in lysis buffer (20 mM Hepes, 0.8 M sorbitol, 50 mM potassium acetate pH 7.0, 2 mM EDTA) and disrupted in a dounce homogenizer on ice. The resulting lysate was subjected to differential centrifugation as previously described^12^, resulting in four subcellular fractions: cell debris and major cell wall fraction (300 g pellet); crude membrane fraction (P13; 13,000 g) containing endosomal membranes, Golgi, ER, plasma membrane and cell wall remnants that are not completely removed by zymolyase treatment; vesicle fraction (P100; 100,000 g pellet) containing endosomal membranes, Golgi membranes and vesicles; cytosolic fraction (S100; 100,000 g supernatant). Cytoplasmic proteins in the 100,000 g supernatant were concentrated by precipitation with 70% ethanol. All samples were separated by SDS-PAGE under reducing conditions, blotted onto PVDF membranes and probed with antibodies directed against various marker proteins (Supplementary Table S3).

### Purification and cellular uptake of Kar2p

Kar2p expression and purification was conducted as previously described^45^. Briefly, *KAR2* without signal sequence was amplified from genomic *S*. *cerevisiae* DNA beginning with the alanine at amino acid position 43. Primers contained an N-terminal His^+^_6_-tag, a *Nde*I site (5′) and a *Not*I site (3′) (Supplementary Table S2). For expression in *E*. *coli*, *KAR2* was inserted into pET-24a^(+)^ as *Nde*I/*Not*I fragment. *E*. *coli* BL21 (DE3) cells expressing pET-24a(+) were grown at 28 °C in LB plus medium containing 31.25 μg/ml kanamycin to an OD_600_ of 0.8. After induction with IPTG (1 mM) for 2.5 h cells were harvested and washed once with water. The cell pellet from 1 l culture was resuspended in buffer D (50 mM Hepes pH 6.8, 0.4 M KOAc, 5 mM MgOAc_2_, 3.5 mM β-mercaptoethanol, 2 mM imidazole pH 7.0) plus protease inhibitor cocktail complete without EDTA (Roche). Cells were disrupted via sonification. After centrifugation (15,000 rpm, 20 min), the supernatant was applied onto a 5 ml Nickel-column (His Trap, GE Healthcare) and washed with buffer D. After washing with 20 mM Hepes (pH 6.8, 1 M KOAc, 0.1% Triton X-100, 5 mM MgOAc_2_, 3.5 mM β-mercaptoethanol, 10 mM imidazole, pH 7.0) and buffer E (20 mM Hepes pH 6.8, 0.25 M KOAc, 5 mM MgOAc_2_, 3.5 mM β-mercaptoethanol, 25 mM imidazole, pH 7.0) proteins were eluted in buffer E containing 250 mM imidazole. The eluate was transferred into B88 buffer (20 mM Hepes pH 6.8, 0.15 M KOAc, 0.25 M sorbitol, 5 mM MgOAc_2_) using gel filtration chromatography (Sephadex G-25 fine, Pharmacia) and subsequently concentrated using Vivaspin concentrators (30 kDa, Sartorius). After the addition of 10% glycerol and sorbitol to a final concentration of 0.8 M, the solution was sterile-filtrated and aliquots were frozen in liquid nitrogen.

Erd2p-mediated uptake of exogenously applied Kar2p was assayed in OP96U oxoplates (PreSens) as previously described^46^. Briefly, 1.8 × 10^7^ yeast cell spheroplasts per well were resuspended in McIlvaine buffer (pH 4.7, 0.8 M sorbitol, 10 mM glucose, 10 mM CaCl_2_) and incubated for 30 min at 30 °C and 120 rpm in the presence of increasing amounts of Kar2p or bovine serum albumin (BSA; negative control). Oxygen concentration was measured every 20 min over a time window of 16 h and pO_2_ values were calculated as described^47^. Each sample measurement was performed in triplicate with a fluorescence reader (Fluoroskan Ascent Labsystems).

### Internalization of Kar2p

Kar2p internalization was determined in wild-type and Δ*end3* yeast cultures (50 ml each) grown over night to exponential phase (1 × 10^7 ^cells/ml) and harvested for 5 min at 8,000 rpm. Thereafter, cells were converted into spheroplasts by zymolyase treatment for 90 min as described in^7^. After three washing steps with incubation buffer (10% v/v McIlvaine pH 4.7, 0.8 M sorbitol, 10 mM CaCl_2_, 10 mM glucose), cells were resuspended in 10 ml incubation buffer and incubated in the presence of purified and N-terminally (His)_6_-tagged Kar2p (9 μg/ml) for 2 h at 30 °C. Thereafter, cells were harvested for 10 min at 2,000 rpm (4 °C), subsequently washed four times with incubation buffer and finally lysed in 150 μl SDS sample buffer supplemented with a protease inhibitor cocktail (Roche) by using a beat beater (Precellys Evolution, Peqlab). After 5 min at 95 °C, samples were cleared by centrifugation (15 min, 13,000 rpm, 4 °C) and supernatants were subjected to SDS-PAGE and immunoblotting. Anti-His antibodies were used to detect internalized Kar2p. Rpn12p served as loading control and was detected via anti-Rpn12 antibodies.

### Killer toxin internalization assay

K28 internalization was assayed from yeast cultures (50 ml) grown over night to exponential phase (1 × 10^7 ^cells/ml) and harvested for 5 min at 8,000 rpm. Thereafter, cells were resuspended in 10 ml incubation buffer (10% v/v McIlvaine pH 4.7, 0.8 M sorbitol, 10 mM CaCl_2_, 10 mM glucose) and 2 ml aliquots were incubated in the presence of V5-tagged K28 (3 μg/ml) for 3 h at 20 °C. Thereafter, cells were harvested for 2 min at 10,000 rpm (4 °C), pellets were subsequently washed twice with McIlvaine buffer (0.1 M citrate, 0.2 M Na_2_HPO_4_, 0.5 M NaCl, pH 4.7) and PK buffer (10 mM Tris/HCL pH 7.5, 20 mM CaCl_2_, 50% glycerol) and finally resuspended in 1 ml PK buffer. To remove remaining cell-bound toxin, samples were incubated for 24 h at 4 °C in the presence of 1.2 mg proteinase K (20 mg/ml stock solution in PK buffer). The reaction was terminated by adding 2.8 mM freshly prepared PMSF (100 mM, in ethanol) and incubation for 30 min at 4 °C. Cells were centrifuged for 2 min at 10,000 rpm, washed twice with McIlvaine buffer and lysed in lysis buffer (10 mM EDTA, 10 mM MOPS pH 6.8, 8 M Urea, 1% SDS, 25 mg/ml NEM) supplemented with a protease inhibitor cocktail (Roche) by using a beat beater (Precellys Evolution, Peqlab). After centrifugation at 13,000 rpm for 5 min at 4 °C, proteins in the cell lysate were precipitated with TCA (10%) over night. Samples were cleared by centrifugation (30 min, 13,000 rpm, 4 °C) and pellets were resuspended in reducing SDS sample buffer and subjected to SDS-PAGE and immunoblotting.

### Western analysis

SDS-PAGE was performed under non-reducing conditions in 10% Tris-Tricine gels using a buffer system according to Schagger & von Jagow^48^. Semi-dry blotting onto PVDF membranes was carried out in transfer buffer (25 mM Tris, 190 mM glycin, 0.1% SDS, 20% methanol). For GFP uptake studies, blots were incubated with antibodies against GFP and different marker proteins. For colorimetric signal detection, primary antibodies were visualized with anti-mouse or anti-rabbit conjugated to alkaline phosphatase and NBT/BCIP solution (Roche). For K28 internalization studies, blots were incubated with primary anti-V5 and anti-Pgk1p (phosphoglycerate kinase) and visualized with secondary HRP-coupled anti-mouse IgG. After incubation with Western lightning Plus ECL (PerkinElmer), signals were detected with ChemiDoc XRS (BioRad). Antibody dilutions are described in Supplementary Table S3.

### Confocal and TIRF microscopy

Single- and two-color imaging of mCherry/eGFP-labeled proteins and FM4-64 staining was performed on a Zeiss confocal microscope LSM 510 META. Cells expressing Can1-mCherry fusions were cultivated in medium with a limited arginine content (2 μg/ml) to avoid increased transport to the vacuole^49^. Cells were embedded in 1% low melting agarose. Two color images of GFP and mCherry fusion proteins or FM4-64 were obtained by confocal fluorescence microscopy using a Zeiss LSM 510 META (488 nm excitation, HFT 488 and NFT 490 beam splitter, BP 500-530 filter; 514 nm excitation, HFT 514 and NFT 545 beam splitter, LP 560 filter). FM4-64 staining (Molecular Probes, Invitrogen) was performed as previously described^50^. Total internal reflection fluorescence (TIRF) microscopy was performed by using a TIRF setup equipped with a Quant EM camera, a 100x/1.45 NA Plan Apochromat TIRF objective, and a multi-line Argon Laser 1885F12 emitting at 488 nm.

### Caging diameter determination

Yeast wild-type strain BY4742 or Δ*end3* cells carrying pRS316 Erd2GFP and grown in synthetic galactose medium were resuspended in PBS and adhered to the surface of a concanvalin A coated (2 mg/ml) coverslip. TIRF microscopy was carried out as described previously^51^. TIRF setup was equipped with a Quant EM camera (Roper Scientific) and a 100x/1.45 NA Plan Apochromat TIRF objective (Olympus Optical), a TILL-TIRF condenser (TILL-Photonics) and the multi-line Argon Laser 1885F12 (Spectra-Physics) emitting at 488 nm. Pixel size was 160 nm. The experimental penetration depth was 245 ± 53 nm (SD, n = 6) determined using a 1 μm fluorescent bead (Invitrogen) as described^52^. Acquisition time was 2 min at 10 Hz; 180 cells were analysed. Erd2p-GFP vesicles were tracked using a software developed with Labview (LV National Instruments) which calculates the centroid position of the vesicle on each image. Precise subpixel accuracy was achieved using this tracking routine. Caging diameter (CD) was obtained using a routine written in Igor (WaveMetrics) as described previously^52^. Briefly, for each trajectory, a sliding window of 1 s was set in which the distance between the first position occupied by the vesicle and all the other positions within this time window was measured, and the maximum reached distance (CD) was determined. Thereby, vesicles displaying small CD correspond to vesicles that move very little, while vesicles with large CD move in a large area. This procedure was applied to all of the positions occupied by the vesicles throughout the 2 min of acquisition. Vesicles visualized for <0.3 s were omitted.

### Data analysis and statistics

Statistical analysis was carried out in Excel (Microsoft). All pooled data were given as mean values ± SD, and statistical significance was assessed by unpaired Student’s t-test analysis based on biological replicates and at sample sizes of n > 10 (*P < 0.05; **P < 0.01; ***P < 0.001).

## Additional Information

**How to cite this article**: Becker, B. *et al*. H/KDEL receptors mediate host cell intoxication by a viral A/B toxin in yeast. *Sci. Rep.* **6**, 31105; doi: 10.1038/srep31105 (2016).

## Supplementary Material

Supplementary Information
