# Supplementary material for: H/KDEL receptors mediate host cell intoxication by a viral A/B toxin in yeast
Source: Sci Rep. 2016 Aug 5;6:31105. doi: 10.1038/srep31105 (PMC4974620; doi:10.1038/srep31105)
Supplement: Supplementary Information [file srep31105-s1.pdf]

Supplementary Information

H/KDEL receptors mediate host cell intoxication by a viral A/B toxin in yeast

Björn Becker, Andrea Blum, Esther Gießelmann, Julia Dausend, Domenik Rammo, Nina C. Müller, Emilia Tschacksch, Miriam Steimer, Jenny Spindler, Ute Becherer, Jens Rettig, Frank Breinig, and Manfred J. Schmitt*

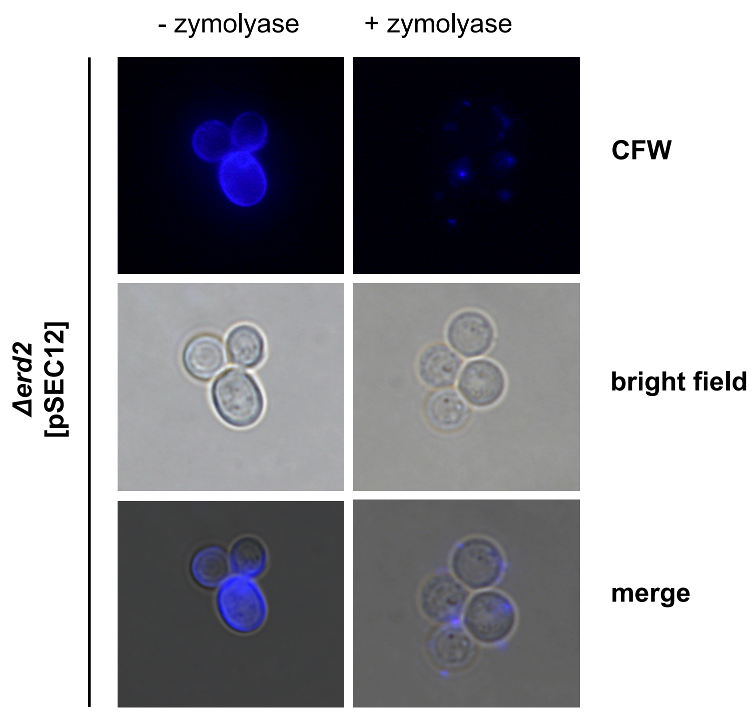

Figure S1: Calcofluor white staining of intact yeast and spheroplasts to detect remnant cell wall chitin after zymolyase treatment. Yeast cells lacking Erd2p (Δ*erd2* [pSEC12]) were treated with (+) or without (-) zymolyase and subsequently stained with calcofluor white (CFW).

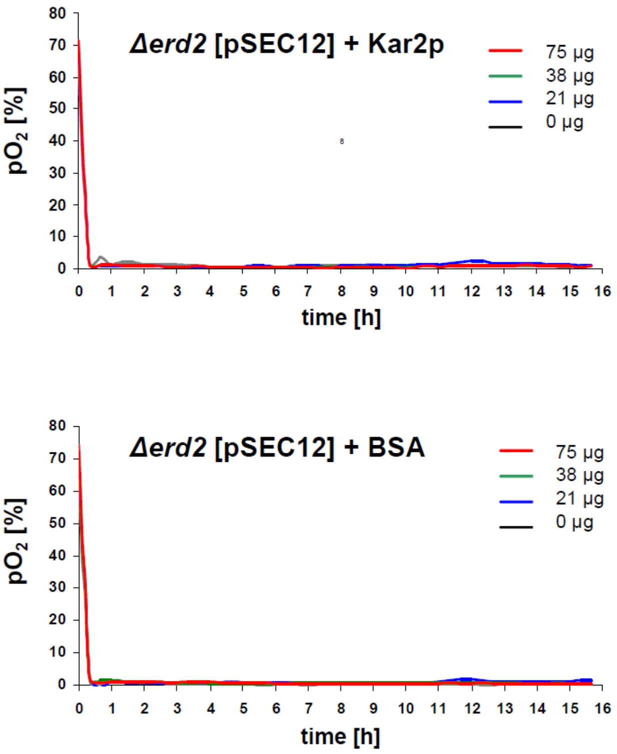

Figure S2: Cell growth of yeast spheroplasts from an HDEL receptor defective mutant in the presence of extracellular Kar2p. Dissolved oxygen content in the culture supernatant of spheroplasts from *Δerd2* cells (kept alive by overexpression of *SEC12* from an episomal plasmid (pSEC12)) was measured in the presence of increasing amounts of Kar2p or bovine serum albumin (BSA) as negative control. Each experiment was performed in triplicate (n = 3); shown is the mean average.

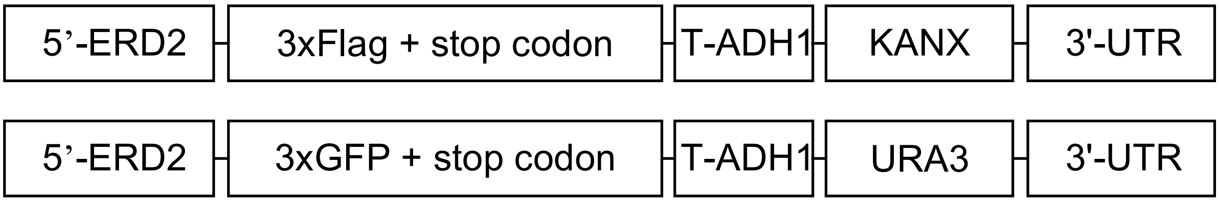

Figure S3: Schematic outline of synthetic DNA constructs used for chromosomal GFP or Flag tagging of Erd2p via homologous recombination. The entire sequence of both DNA constructs is given in Appendix S1. (5'-ERD2, 200 bp of the 3'-end of *ERD2*; 3xGFP, three copies of yeast enhanced GFP; 3xFlag, three copies of a Flag tag; T-ADH1, transcriptional terminator of the yeast alcohol dehydrogenase gene *ADH1*; URA3, expression cassette of the yeast *URA3* gene; KANX, kanamycin resistance cassette; 3'-UTR, untranslated region of *ERD2*).

Table S1. Yeast strains used in this study

***S. cerevisiae* strain**	**Genotype**	**Reference**
Δerd2 (YA12)	MATα *ade2 ade3 his3-Δ200 leu2-3, 112 ura3-52 TRP1 erd2-ΔNco* [pY*SEC12*]	[1]
SEY6210	MATα *ura3-52 leu2-3, 112 his3-Δ200 trp1-Δ901 lys2-801 suc2-Δ9*	[1]
ERD2 [pERD2]	SEY6210 [pERD2] (pERD2 = pYSCE)	This study<br>pYSCE: H.Pelham
BY4742	MATα *his3Δ1 leu2Δ0 lys2Δ0 ura3Δ0*	OpenBiosystems
*Δ*end3	MATα *his3Δ1 leu2Δ0 lys2Δ0 ura3Δ0 Δend3*	OpenBiosystems
cdc25-2	MATα *ura3 leu2 trp1 hisΔ200 ade2-101 cdc25-2*	[2]
KAR2 (RSY255)	MATa *ura3-52 leu2-3, -112*	[3]
$kar2^{ts}$ (RSY475)	MATa *ura3-52 leu2-3, -112 kar2-159*	[3]
end3 (RH266-1D)	MAT*α end3 ura3 leu2 his4 bar1-1*	[4]
YA ERD2-ΔC	MATα *ade2 ade3 his3-Δ200 leu2-3, 112 ura3-52 TRP1 erd2-ΔNco* [pRS315 *ERD2- ΔC*]; *ΔC*: deletion of the C-terminal 39 amino acids	This study
YA ERD2	MATα *ade2 ade3 his3-Δ200 leu2-3, 112 ura3-52 TRP1 erd2-ΔNco* [pRS315 *ERD2*]	This study
BY4742 *ERD2mRAS*	MATα *his3Δ1 leu2Δ0 lys2Δ0 ura3Δ0* [pRS316 *ERD2mRAS*]	This study
BY4742 *ICE2mRAS*	MATα *his3Δ1 leu2Δ0 lys2Δ0 ura3Δ0* [pRS316 *ICE2mRAS*]	This study
BY4742 *EMP47mRAS*	MATα *his3Δ1 leu2Δ0 lys2Δ0 ura3Δ0* [pRS316 *EMP47mRAS*]	This study
YA ERD2.1	MATα *ade2 ade3 his3-Δ200 leu2-3, 112 ura3-52 TRP1 erd2-ΔNco* [pRS315 *ERD2.1*] = mammalian KDELR1	This study
YA ERD2.2	MATα *ade2 ade3 his3-Δ200 leu2-3, 112 ura3-52 TRP1 erd2-ΔNco* [pRS315 *ERD2.2*] = mammalian KDELR2	This study
YA ERD2.3	MATα *ade2 ade3 his3-Δ200 leu2-3, 112 ura3-52 TRP1 erd2-ΔNco* [pRS315 *ERD2.3*] = mammalian KDELR3	This study
BY4742 *ERD2yGFP*	MATα *his3Δ1 leu2Δ0 lys2Δ0 ura3Δ0* [pRS316 *ERD2yGFP*]	This study
BY4742 *Can1-mCherry + ERD2yGFP*	MATα *his3Δ1 leu2Δ0 lys2Δ0 ura3Δ0* [pRS315 Can1-mCherry] + [pRS316 *ERD2yGFP*]	This study
BY4742 *Pil1-mCherry + ERD2yGFP*	MATα *his3Δ1 leu2Δ0 lys2Δ0 ura3Δ0* [pRS315 Pil1-mCherry] + [pRS316 *ERD2yGFP*]	This study
BY4742 *Sur7-mCherry + ERD2yGFP*	MATα *his3Δ1 leu2Δ0 lys2Δ0 ura3Δ0* [pRS315 Sur7-mCherry] + [pRS316 *ERD2yGFP*]	This study
BY4742 *Anp1-mCherry + ERD2yGFP*	MATα *his3Δ1 leu2Δ0 lys2Δ0 ura3Δ0* [pRS315 Anp1-mCherry] + [pRS316 *ERD2yGFP*]	This study
BY4742 *ERD2-3xyGFP*	MATα *his3Δ1 leu2Δ0 lys2Δ0 ura3Δ0 erd2::ERD2-3xyGFP-URA3*	This study
BY4742 *ERD2-3xFLAG*	MATα *his3Δ1 leu2Δ0 lys2Δ0 ura3Δ0 erd2::ERD2-3xFlag-KANX*	This study

1. Hardwick, K. G., Boothroyd, J. C., Rudner, A. D. & Pelham, H. R. Genes that allow yeast cells to grow in the absence of the HDEL receptor. Embo J 11, 4187-95 (1992).
2. Broder, Y. C., Katz, S. & Aronheim, A. The ras recruitment system, a novel approach to the study of protein-protein interactions. Curr Biol 8, 1121-4 (1998).
3. Wuestehube, L. J. et al. New mutants of Saccharomyces cerevisiae affected in the transport of proteins from the endoplasmic reticulum to the Golgi complex. Genetics 142, 393-406 (1996).
4. Raths, S., Rohrer, J., Crausaz, F. & Riezman, H. end3 and end4: two mutants defective in receptor-mediated and fluid-phase endocytosis in Saccharomyces cerevisiae. J Cell Biol 120, 55-65 (1993).

Table S2. Primers used in this study (restriction sites are shown in lower case letters)

**Primer**	**5'-3' Sequence**
3' ANP1	agtactGTTTCTATCAGGGTCGAAGTCTAATGGAACTTC
3' CAN1	agtactTGCTACAACATTCCAAAATTTGTCCCAAAAAAG
3' PIL1	agtactAGCTGTTGTTTGTTGGGGAAGAGACTC
3' SUR7	agtactAACAGAGACATCGTCCGGGCGCTCGTGTG
3' CYC TT	gagctcGCAGCTTGCAAATTAAAGCCTTCGAG
3' ERD2GFP	GAGGAAAGGGTTTCAAACTGCCAAAAgtcgacATGTCTAAAGGTGAAGAATTATTCACTGGTGTTG
3' GFP	gaattcTTACTTGTACAGCTCGTCCATGCCGAG
3' GFP^{HDEL}	gaattcTTATAGCTCATCGTGCTTGTACAGCTCGTCCATGCCGAGAGGAGAG
3' mCherry	ggatccgtcgacTTACTTGTACAGCTCGTCCATGCCG
3' mRAS	ggatccTTACTTGCAGCTCATGCAGCCG
3' P GAL1	gaattcctcgagGGTTTTTTCTCCTTGACGTTAAAGTATAGAGGTATATTAACAATTTTTTGTTGATACTTTTATTACATT TGAATAAGAAG
3' ICE2	ggatccactagtACTACCAGAACCTATTAATTCTGTAGCGTATACC
3' ERD2	gtcgactctagaactagtagatctTTATTTTGGCAGTTTGAAACCCTTTCCTCT
3'ERD2.1 (human)	ggatccgtcgacCTATGCCGGCAAACTCAACTTCTTCC
3'ERD2.2 (human)	ggatccgtcgacTTATGCTGGCAAACTGAGCTTCTTTCC
3'ERD2.3 (human)	ggatccgtcgacTCAGATTGGCATTGGAAGACTTAACTTCTTTC
3' KAR2	gtcgacgcggccgcCTACAATTCGTCGTGTTCGAAATAATCACCATC
3' yEGFP	agatctgtcgacTTATTTGTACAATTCATCCATACCATGGGTAATACC
5' CAN1	ctcgagATGACAAATTCAAAAGAAGACGCCGAC
5' ANP1	ctcgagATGAAGTATAATAACAGAAAACTCTCGTTCAACCCTAC
5' PIL1	ctcgagATGCACAGAACTTACTCTTTAAGAAATTCCAGG
5' SUR7	ctcgagATGGTTAAGGTCTGGAATATAGTACTACGTCTGGTAG
5' CYC TT	tctagagtcgacggatcccATGTAATTAGT
5' ERD2GFP	GAGGAAAGGGTTTCAAACTGCCAAAAgtcgacATGTC TAAAGGTGAAGAATTATTCACTGGTGTTG
5' GFP	catatgGTGAGCAAGGGCGAGGAGGTGTTC
5' mCherry	ctcgagtctagaactagtATGGTGAGCAAGGGCGAGGAG
5' mRAS	actagtATGACGGAATATAAGCTGGTGGTGGTG
5' ICE2	ctcgaggaatccATGACCAGTTTGTCCAAAAGCTTCATG
5' P GAL1	gtcgacgcggccgcACGGATTAGAAGCCGCCGAGC
5' ERD2	ctcgaggaattcATGAATCCGTTTAGAATCTTAGGTGATTTATCACAT
5'ERD2.1 (human)	gagctcgaattcATGAATCTCTTCCGATTCCTGGGAGAC
5'ERD2.2 (human)	gagctcgaattcATGAACATTTTCCGGCTGACTGGG
5'ERD2.3 (human)	gagctcgaattcATGAACGTGTTCCGAATCCTCGG

Table S3. Antibodies used in this study

Antibody	Dilution	Source
anti-PMA1, rabbit	1 : 1,000	Ralf Kölling
anti-PDI, rabbit	1 : 10,000	Randy Schekman
anti-Pep12, mouse	1 : 200	Molecular Probes
anti-Vps10, mouse	1 : 500	Molecular Probes
anti-PGK, mouse	1 : 250	Molecular Probes
anti-Sec61, rabbit	1 : 5,000	Randy Schekman
anti-Rpn12, rabbit	1 : 2,000	Karin Römisch
anti-Rabbit-AP	1 : 3,000	Sigma
anti-Mouse-AP	1 : 3,000	Sigma
anti-Mouse-HRP	1 : 13,333	Sigma
anti-Rabbit-HRP	1 : 10,000	Sigma
anti-GFP, mouse	1 : 2,000	Roche
anti-V5, mouse	1 : 1,000	AbD SeroTec
anti-FLAG M2, mouse	1 : 1,000	Sigma

Appendix S1.

(a) ERD2_3xGFP-URA sequence (5'-3' direction) used for homologous recombination and chromosomal tagging of Erd2p with three C-terminal copies of yGFP.

GAATTCACTGTTCATTATATTTTTGCCATGGGATTATACAGAGCATTGTATATTCCTAACTGGATTTGGAGGTACAGCACGGAAGATAAAAAATTGGACAAGATTGCCTTCTTCGCGGGACTTTT
GCAAACTCTGTTGTACTCTGATTTCTTTTACATTTACTACACTAAAGTCATCAGAGGAAAGGGTTTCAAACTGCCAAAAGGAGGCGGTTCTAAAGGTGAAGAATTATTCACTGGTGTTGTCCCAA
TTTTGGTTGAATTAGATGGTGATGTTAATGGTCACAAATTTTCTGTCTCCGGTGAAGGTGAAGGTGATGCTACTTACGGTAAATTGACCTTAAAATTTATTTGTACTACTGGTAAATTGCCAGTT
CCATGGCCAACCTTAGTCACTACTTTCGGTTATGGTGTTCAATGTTTTGCTAGATACCCAGATCATATGAAACAACATGACTTTTTCAAGTCTGCCATGCCAGAAGGTTATGTTCAAGAAAGAACT
ATTTTTTTCAAAGATGACGGTAACTACAAGACCAGAGCTGAAGTCAAGTTTGAAGGTGATACCTTAGTTAATAGAATCGAATTAAAAGGTATTGATTTTAAAGAAGATGGTAACATTTTAGGTCA
CAAATTGGAATACAACTATAACTCTCACAATGTTTACATCATGGCTGACAAACAAAAGAATGGTATCAAAGTTAACTTCAAAATTAGACACAACATTGAAGATGGTTCTGTTCAATTAGCTGACC
ATTATCAACAAAATACTCCAATTGGTGATGGTCCAGTCTTGTTACCAGACAACCATTACTTATCCACTCAATCTGCCTTATCCAAAGATCCAAACGAAAAGAGAGACCACATGGTCTTGTTAGAAT
TTGTTACTGCTGCTGGTATTACCCATGGTATGGATGAATTGTACAAATCTAAAGGTGAAGAATTATTCACTGGTGTTGTCCCAATTTTGGTTGAATTAGATGGTGATGTTAATGGTCACAAATTT
TCTGTCTCCGGTGAAGGTGAAGGTGATGCTACTTACGGTAAATTGACCTTAAAATTTATTTGTACTACTGGTAAATTGCCAGTTCCATGGCCAACCTTAGTCACTACTTTCGGTTATGGTGTTCAA
TGTTTTGCTAGATACCCAGATCATATGAAACAACATGACTTTTTCAAGTCTGCCATGCCAGAAGGTTATGTTCAAGAAAGAACTATTTTTTTCAAAGATGACGGTAACTACAAGACCAGAGCTGA
AGTCAAGTTTGAAGGTGATACCTTAGTTAATAGAATCGAATTAAAAGGTATTGATTTTAAAGAAGATGGTAACATTTTAGGTCACAAATTGGAATACAACTATAACTCTCACAATGTTTACATCA
TGGCTGACAAACAAAAGAATGGTATCAAAGTTAACTTCAAAATTAGACACAACATTGAAGATGGTTCTGTTCAATTAGCTGACCATTATCAACAAAATACTCCAATTGGTGATGGTCCAGTCTTG
TTACCAGACAACCATTACTTATCCACTCAATCTGCCTTATCCAAAGATCCAAACGAAAAGAGAGACCACATGGTCTTGTTAGAATTTGTTACTGCTGCTGGTATTACCCATGGTATGGATGAATTG
TACAAATCTAAAGGTGAAGAATTATTCACTGGTGTTGTCCCAATTTTGGTTGAATTAGATGGTGATGTTAATGGTCACAAATTTTCTGTCTCCGGTGAAGGTGAAGGTGATGCTACTTACGGTAA
ATTGACCTTAAAATTTATTTGTACTACTGGTAAATTGCCAGTTCCATGGCCAACCTTAGTCACTACTTTCGGTTATGGTGTTCAATGTTTTGCTAGATACCCAGATCATATGAAACAACATGACTT
TTTCAAGTCTGCCATGCCAGAAGGTTATGTTCAAGAAAGAACTATTTTTTTCAAAGATGACGGTAACTACAAGACCAGAGCTGAAGTCAAGTTTGAAGGTGATACCTTAGTTAATAGAATCGAAT
TAAAAGGTATTGATTTTAAAGAAGATGGTAACATTTTAGGTCACAAATTGGAATACAACTATAACTCTCACAATGTTTACATCATGGCTGACAAACAAAAGAATGGTATCAAAGTTAACTTCAAA
ATTAGACACAACATTGAAGATGGTTCTGTTCAATTAGCTGACCATTATCAACAAAATACTCCAATTGGTGATGGTCCAGTCTTGTTACCAGACAACCATTACTTATCCACTCAATCTGCCTTATCC
AAAGATCCAAACGAAAAGAGAGACCACATGGTCTTGTTAGAATTTGTTACTGCTGCTGGTATTACCCATGGTATGGATGAATTGTACAAATAAGCGAATTTCTTATGATTTATGATTTTTATTAT
TAAATAAGTTATAAAAAAAATAAGTGTATACAAATTTTAAAGTGACTCTTAGGTTTTAAAACGAAAATTCTTATTCTTGAGTAACTCTTTCCTGTAGGTCAGGTTGCTTTCTCAGGTATAGTATG
AGGTCGCTCTTATTGACCACACCGACATGGAGGCCCAGAATACCCTCCTTGACAGTCTTGACGTGCGCAGCTCAGGGGCATGATGTGACTGTCGCCCGTACATTTAGCCCATACATCCCCATGTATA
ATCATTTGCATCCATACATTTTGATGGCCGCACGGCGCGAAGCAAAAATTACGGCTCCTCGCTGCAGACCTGCGAGCAGGGAAACGCTCCCCTCACAGACGCGTTGAATTGTCCCCACGCCGCGCCC
CTGTAGAGAAATATAAAAGGTTAGGATTTGCCACTGAGGTTCTTCTTTCATATACTTCCTTTTAAAATCTTGCTAGGATACAGTTCTCACATCACATCCGAACATAAACAACCATGTCGAAAGCTA
CATATAAGGAACGTGCTGCTACTCATCCTAGTCCTGTTGCTGCCAAGCTATTTAATATCATGCACGAAAAGCAAACAAACTTGTGTGCTTCATTGGATGTTCGTACCACCAAGGAATTACTGGAGT
TAGTTGAAGCATTAGGTCCCAAAATTTGTTTACTAAAAACACATGTGGATATCTTGACTGATTTTTCCATGGAGGGCACAGTTAAGCCGCTAAAGGCATTATCCGCCAAGTACAATTTTTTACTCT
TCGAAGACAGAAAATTTGCTGACATTGGTAATACAGTCAAATTGCAGTACTCTGCGGGTGTATACAGAATAGCAGAATGGGCAGACATTACGAATGCACACGGTGTGGTGGGCCCAGGTATTGTT
AGCGGTTTGAAGCAGGCGGCGGAAGAAGTAACAAAGGAACCTAGAGGCCTTTTGATGTTAGCAGAATTGTCATGCAAGGGCTCCCTAGCTACTGGAGAATATACTAAGGGTACTGTTGACATTGC
GAAGAGCGACAAAGATTTTGTTATCGGCTTTATTGCTCAAAGAGACATGGGTGGAAGAGATGAAGGTTACGATTGGTTGATTATGACACCCGGTGTGGGTTTAGATGACAAGGGAGACGCATTGG
GTCAACAGTATAGAACCGTGGATGATGTGGTCTCTACAGGATCTGACATTATTATTGTTGGAAGAGGACTATTTGCAAAGGGAAGGGATGCTAAGGTAGAGGGTGAACGTTACAGAAAAGCAGGC
TGGGAAGCATATTTGAGAAGATGCGGCCAGCAAAACTAATCAGTACTGACAATAAAAAGATTCTTGTTTTCAAGAACTTGTCATTTGTATAGTTTTTTTATATTGTAGTTGTTCTATTTTAATCA
AATGTTAGCGTGATTTATATTTTTTTTCGCCTCGACATCATCTGCCCAGATGCGAAGTTAAGTGCGCAGAAAGTAATATCATGCGTCAATCGTATGTGAATGCTGGTCGCTATACTGAACCTGTTT
TAAATAGTGTTCGCAGCAATATATACAAATTTCAATATATCAATGCTATACCAAAAGAAAAAAAAAATACATAAAATTGATGTTGATATTGGTCTATTTCTTTTTATACTATGATATGTATGCAT
TAATCTCTTTTTAGCTCATAAAATTCTTTCCTTACACCATCCTTGGTGACAATAAGGATTTCCAGCCGAATTC

(b) ERD2_3xFlag-KANX sequence (5'-3' direction) used for homologous recombination and chromosomal tagging of Erd2p with three C-terminal copies of a Flag-tag.

ACTGTTCATTATATTTTTGCCATGGGATTATACAGAGCATTGTATATTCCTAACTGGATTTGGAGGTACAGCACGGAAGATAAAAAATTGGACAAGATTGCCTTCTTCGCGGGACTTTTGCAAAC
TCTGTTGTACTCTGATTTCTTTTACATTTACTACACTAAAGTCATCAGAGGAAAGGGTTTCAAACTGCCAAAAGGAGGCGGTGACTACAAGGATGACGATGACAAGGATTATAAAGACGATGACG
ATAAAGATTACAAAGATGATGATGATAAGTAAGCGAATTTCTTATGATTTATGATTTTTATTATTAAATAAGTTATAAAAAAAATAAGTGTATACAAATTTTAAAGTGACTCTTAGGTTTTAAA
ACGAAAATTCTTATTCTTGAGTAACTCTTTCCTGTAGGTCAGGTTGCTTTCTCAGGTATAGTATGAGGTCGCTCTTATTGACCACACCGACATGGAGGCCCAGAATACCCTCCTTGACAGTCTTGA
CGTGCGCAGCTCAGGGGCATGATGTGACTGTCGCCCGTACATTTAGCCCATACATCCCCATGTATAATCATTTGCATCCATACATTTTGATGGCCGCACGGCGCGAAGCAAAAATTACGGCTCCTCG
CTGCAGACCTGCGAGCAGGGAAACGCTCCCCTCACAGACGCGTTGAATTGTCCCCACGCCGCGCCCCTGTAGAGAAATATAAAAGGTTAGGATTTGCCACTGAGGTTCTTCTTTCATATACTTCCTT
TTAAAATCTTGCTAGGATACAGTTCTCACATCACATCCGAACATAAACAACCATGGGTAAGGAAAAGACTCACGTTTCGAGGCCGCGATTAAATTCCAACATGGATGCTGATTTATATGGGTATA

AATGGGCTCGCGATAATGTCGGGCAATCAGGTGCGACAATCTATCGATTGTATGGGAAGCCCGATGCGCCAGAGTTGTTTCTGAAACATGGCAAAGGTAGCGTTGCCAATGATGTTACAGATGAG
ATGGTCAGACTAAACTGGCTGACGGAATTTATGCCTCTTCCGACCATCAAGCATTTTATCCGTACTCCTGATGATGCATGGTTACTCACCACTGCGATCCCCGGCAAAACAGCATTCCAGGTATTA
GAAGAATATCCTGATTCAGGTGAAAATATTGTTGATGCGCTGGCAGTGTTCCTGCGCCGGTTGCATTCGATTCCTGTTTGTAATTGTCCTTTTAACAGCGATCGCGTATTTCGTCTCGCTCAGGCG
CAATCACGAATGAATAACGGTTTGGTTGATGCGAGTGATTTTGATGACGAGCGTAATGGCTGGCCTGTTGAACAAGTCTGGAAAGAAATGCATAAGCTTTTGCCATTCTCACCGGATTCAGTCGT
CACTCATGGTGATTTCTCACTTGATAACCTTATTTTTGACGAGGGGAAATTAATAGGTTGTATTGATGTTGGACGAGTCGGAATCGCAGACCGATACCAGGATCTTGCCATCCTATGGAACTGCCT
CGGTGAGTTTTCTCCTTCATTACAGAAACGGCTTTTTCAAAAATATGGTATTGATAATCCTGATATGAATAAATTGCAGTTTCATTTGATGCTCGATGAGTTTTTCTAATCAGTACTGACAATAA
AAAGATTCTTGTTTTCAAGAACTTGTCATTTGTATAGTTTTTTTATATTGTAGTTGTTCTATTTTAATCAAATGTTAGCGTGATTTATATTTTTTTTCGCCTCGACATCATCTGCCCAGATGCGAA
GTTAAGTGCGCAGAAAGTAATATCATGCGTCAATCGTATGTGAATGCTGGTCGCTATACTGAACCTGTTTTAAATAGTGTTCGCAGCAATATATACAAATTTCAATATATCAATGCTATACCAAA
AGAAAAAAAAAATACATAAAATTGATGTTGATATTGGTCTATTTCTTTTTATACTATGATATGTATGCATTAATCTCTTTTTAGCTCATAAAATTCTTTCCTTACACCATCCTTGGTGACAATAA
GGATTTCCAGCC

(c) EMP47 sequence used for the construction of EMP47-mRAS

CTCGAGATGATGATGTTAATTACTATGAAAAGTACAGTACTGTTGAGTGTTTTTACCGTCTTAGCGACATGGGCTGGATTGCTAGAAGCTCATCCATTGGGTGACACTTCAGATGCATCCAAATT
AAGCTCAGACTACTCGCTCCCTGATCTCATTAATGCACGTAAAGTGCCCAATAACTGGCAAACTGGAGAACAAGCTAGTCTAGAGGAAGGGAGAATTGTATTGACTTCTAAGCAAAATTCCAAGG
GTTCACTTTGGTTGAAGCAAGGATTCGATTTGAAGGATTCTTTTACTATGGAGTGGACATTTAGGAGTGTTGGTTATTCTGGCCAAACCGACGGTGGCATATCATTTTGGTTTGTTCAAGATTCT
AACGTACCACGCGATAAGCAGTTATACAATGGGCCAGTGAACTATGATGGTTTACAATTATTAGTGGATAACAATGGTCCATTGGGCCCAACACTTCGTGGTCAACTAAATGATGGTCAAAAGCC
TGTAGATAAGACGAAAATCTATGATCAGAGTTTTGCATCTTGTTTGATGGGTTATCAGGATTCCTCCGTTCCTTCCACGATCAGAGTAACTTATGATTTGGAAGACGACAACTTATTAAAAGTTC
AGGTGGACAATAAAGTCTGTTTCCAAACTAGGAAGGTTCGCTTTCCCTCTGGGTCTTACCGTATTGGTGTCACCGCTCAAAATGGAGCAGTGAATAATAATGCAGAGTCTTTTGAAATATTCAAA
ATGCAATTTTTTAATGGCGTGATTGAAGATTCTTTGATCCCTAATGTGAATGCAATGGGTCAGCCAAAACTGATCACCAAATACATTGACCAACAAACCGGCAAAGAAAAATTGATTGAAAAAAC
AGCATTTGACGCTGACAAAGACAAAATTACAAACTATGAATTGTATAAGAAACTGGATAGAGTTGAAGGTAAAATTCTTGCGAACGATATCAATGCTTTAGAAACAAAGCTAAATGATGTCATT
AAGGTCCAACAAGAGCTATTATCATTCATGACTACGATAACTAAACAGCTCTCTTCTAAGCCACCAGCTAATAATGAAAAGGGAACGTCCACCGATGATGCAATCGCTGAGGATAAAGAAAATTT
CAAAGACTTCTTATCAATCAATCAGAAATTGGAGAAAGTCCTGGTTGAACAAGAAAAGTATAGGGAAGCTACCAAACGTCATGGACAAGATGGTCCTCAGGTCGACGAAATTGCCAGAAAACTAA
TGATTTGGTTACTTCCATTAATTTTCATTATGTTGGTTATGGCATATTACACATTCAGAATCAGACAAGAGATCATAAAGACCAAACTGCTAACTAGT
